# Pathological Observations on the Diseases of the Uterus

**Published:** 1840-07

**Authors:** 


Art. X.—*Pathological Observations on the Diseases of the Uterus.* By Robert Lee, m.d., f.r.s., Physician to the British Lying-in Hospital. *With coloured illustrations from original drawings by* Mr. Perry. Part I.—*London*, 1840. Fol. pp. 12. *Four Plates.*

This is the first fasciculus of a work in which Dr. Lee proposes "to illustrate, by cases and coloured plates, the nature, symptoms, and treatment" of the diseases of the uterus and its appendages, arranged under the following heads:—1. Malignant diseases. 2. Fibrous, calcareous, and other tumours. 3. Inflammation and functional diseases. 4. Diseases of the ovaria, &c. 5. Diseases of the gravid uterus. The part before us consists of a series of forty-two cases of malignant disease, in different forms and stages, concisely yet carefully detailed, and four plates—three illustrative of the cases, and one, which seems much out of place here although in itself curious, a drawing of a malformed fœtus described by the author in vol. xxii. of the Med. Chir. Trans. When completed this work will be very valuable, as we presume the author, ere he closes it, will place the results of his anatomical researches and practical experience in the form of general summaries of some length before his readers. At present it can hardly be looked upon as much more than as a treasury of valuable facts in morbid anatomy, rendered indeed mutually illustrative by classification, yet still awaiting the generalizing touch of the author to have their full value educed for the benefit of pathology and practical medicine. We know few more capable of doing this than Dr. Lee; and we trust that it is his purpose to do so. In the meantime we must content ourselves with the following summary appended to the present series of cases, the excellence of which makes us regret its extreme brevity:

"From these cases it will be seen that the fungoid tumour of the uterus, or cauliflower excrescence, scirrhus, carcinoma, and corroding ulcer, are merely different forms of the same malignant disease; that the morbid changes may commence in the mucous and muscular coats of the fundus and body of the uterus, though they are observed most frequently to begin in the orifice and cervix. It may be inferred, also, from these histories that inflammation of the uterus does not give rise to cancer in any form; and that the progress of the disease to a fatal termination is never arrested by those remedies which subdue inflammation. Three of the individuals whose cases have been related were under thirty years of age, three between thirty and forty, sixteen from forty to fifty, and fourteen from fifty to sixty. In ten cases there was either no pain whatever experienced in the uterus, or it was only a slight dull pain or sense of uneasiness within the pelvis. Hemorrhage from the uterus, fœtid discharge of serum, mucus, and pus mixed with blood from the vagina, and sickness at stomach, were the symptoms almost invariably present after ulceration had taken place. The hemorrhage from the uterus was most profuse and the pain slightest in the fungoid form of the disease.

"In the treatment of these cases when the pain was severe, relief was occasionally obtained by leeches applied to the anus, hypogastrium, and groins, and cupping-glasses to the sacrum. In no case was great benefit derived from the application of leeches to the os uteri. The frequent injection of tepid water into the vagina, decoction of poppies, solutions of opium, conium, lead, zinc, nitrate of silver, and chloride of soda, often diminished the fetor of the discharge and soothed the sufferings of the patients. The tepid hip-bath often afforded great relief; and the pain was frequently mitigated by friction with camphorated liniment and laudanum over the loins, lower part of the spine and sacrum, and the whole region of the uterus. The belladonna plaster over the sacrum was only useful in a few cases and for a short period. Excruciating pain was often diminished by an enema of laudanum, and a little warm milk or solution of starch. Solid opium, the liquor opii sedativus, the preparations of morphine, and the extract of hyoscyamus, were the most useful internal remedies.

"Neither the removal of the whole uterus by a surgical operation nor the destruction of its orifice with caustic or other means was considered justifiable in any case." (p. 12.)

It would be injustice to the work and the artist, Mr. Perry, not to call attention to the great beauty of the plates.

